# The use of activated vitamin D and risks of hospitalization for infection and amputation in incident hemodialysis patients in Taiwan: a nationwide population-based cohort study

**DOI:** 10.1186/s12882-020-01988-2

**Published:** 2020-08-06

**Authors:** Jo-Yen Chao, Chung-Yi Li, Ming-Cheng Wang, Yea-Huei Kao Yang

**Affiliations:** 1 Division of Nephrology, Department of Internal Medicine, National Cheng Kung University Hospital, College of Medicine, National Cheng Kung University, Tainan, Taiwan; 2 Institute of Clinical Pharmacy and Pharmaceutical Sciences, College of Medicine, National Cheng Kung University, No.1, University Road, Tainan, 70101 Taiwan; 3 Department of Public Health, College of Medicine, National Cheng Kung University, Tainan, Taiwan

**Keywords:** Hemodialysis, Activated vitamin D, Acute myocardial infarction, Ischemic stroke, Amputation, Infection, Death, Competing risk analysis

## Abstract

**Background:**

Hemodialysis patients have a high risk of mortality. The most common causes of death are cardiovascular disease and infection. The potential hazard or benefit associated with vitamin D use and cardiovascular or infection outcome is poorly characterized.

**Methods:**

We conducted a retrospective observational cohort study by recruiting 52,757 patients older than 20 years from Taiwan National Health Insurance Research Database (NHIRD) who initiated maintenance hemodialysis between 2001 and 2009. Patients who were prescribed activated vitamin D before the 360th day from hemodialysis initiation were defined as vitamin D users. The primary outcome of interest includes occurrence of acute myocardial infarction (AMI), ischemic stroke, lower limb amputation, and hospitalization for infection, respectively, while death events are treated as competing events. We conducted competing risk analysis using subdistribution hazard regression model to estimate subdistribution hazard ratios (SHRs) in relation to various outcomes.

**Results:**

During the median follow-up of 1019 days, the vitamin D users had a lower crude mortality rate, lower incidences of AMI, ischemic stroke, amputation, and hospitalization for infection compared with non-users. Taking into consideration competing events of death, vitamin D users were associated with a lower hazard of lower limb amputation (SHR 0.84 [95% CI, 0.74–0.96]) and hospitalization for infection (SHR 0.90 [95% CI, 0.87–0.94]), but not AMI or ischemic stroke, after adjustment for potential confounders. Subgroup analyses and dose response evaluation both showed a consistent association of activated vitamin D treatment with decreased risk of amputation and infection.

**Conclusion:**

The findings suggest that therapeutic activated vitamin D use in hemodialysis patients may be beneficial for decreasing infection events and amputation, of which the latter is a complication of peripheral vascular disease, rather than reducing major atherosclerotic cardiovascular events such as AMI or ischemic stroke.

## Background

In chronic kidney disease (CKD) and end-stage renal disease (ESRD) patients, vitamin D deficiency and insufficiency were associated with increased risk of death [1–3]. In addition, vitamin D deficiency was associated with increased cardiovascular events and infection [4, 5]. Diabetes, dyslipidemia, derangement of calcium and phosphorus metabolism, vitamin D deficiency, and hyperparathyroidism in CKD all contribute to vascular calcification. Arterial medial calcification is associated with an increased risk of peripheral artery disease (PAD), heart failure, lower limb amputation, and mortality [6, 7].

In practice, activated vitamin D is indicated in CKD or ESRD patients with hypocalcemia and secondary hyperparathyroidism [8]. In observational studies, patients treated with activated vitamin D had a lower all-cause mortality rate, and a lower prevalence of pulmonary congestion, compared with those not treated [9–11]. Effects of activated vitamin D include suppression of renin synthesis, inhibition of vascular calcification, and modification of immune system [12, 13]. The immunomodulatory effects of vitamin D include enhanced production of endogenous antimicrobial peptide cathelicidin by monocytes and regulation of adaptive immune system, especially T cells [14, 15]. Besides, administration of vitamin D in ESRD patients is noted with a lower risk of infection especially airway or pulmonary infections [5, 16].

Our previous analysis showed that activated vitamin D treatment was associated with a reduced all-cause mortality in incident hemodialysis patients [17]. We aim to further assess whether administration of activated vitamin D is associated with reduced risks of acute myocardial infarction (AMI), ischemic stroke, amputation, or infectious events in these patients.

## Methods

### Data source

Taiwan National Health Insurance (NHI) is a single-payer and mandatory enrollment health care program launched in 1995. It comprehensively covered over 23 million residents in Taiwan. The National Health Insurance Research Database (NHIRD) contains registration files and de-identified claim data for reimbursement. The registry of catastrophic illness is a subset of NHI program and contains patients with specific severe disease conditions. In Taiwan, ESRD patients initiating maintenance dialysis are eligible for catastrophic illness certificate (CIC) and thus can be exempted from co-payment for the illness or related conditions. This registry is representative of most, if not all, patients with medically qualified diseases.

All diagnoses in the NHIRD were coded according to the International Classification of Disease, 9th revision, Clinical Modification (ICD-9-CM).

### Study design

We conducted a retrospective cohort study by recruiting all incident hemodialysis patients with issue of CIC of uremia (ICD-9: 585) from January 1, 2001 to June 30, 2009 from NHIRD. Patients who were younger than 20 years, transferred to peritoneal dialysis, with history of malignancy, or those with a graft kidney failure and re-initiated dialysis were excluded. The date of hemodialysis initiation was the cohort entry date. We employed a landmark design and followed the patients from the 360th day after cohort entry, i.e. the landmark time, until the occurrence of events of interest, death, cessation of follow-up, or the end of 2010, which ever came first.

The study protocol was approved by the Institutional Review Board (IRB) of National Cheng Kung University Hospital (IRB number: A-EX-106-038).

### Baseline information and covariates

Age, sex, comorbidities, and medication use were collected from the claims data of ambulatory care and hospital admission within 90 days prior to or after hemodialysis initiation. The baseline comorbidities include diabetes, myocardial infarction, congestive heart failure, cerebrovascular disease, peripheral vascular disease, connective tissue disease, chronic obstructive pulmonary disease, chronic liver disease, peptic ulcer disease, and neoplasia. The baseline medications include antiplatelets, warfarin, cilostazol, statins, oral anti-diabetics agents, insulins, angiotensin converting enzyme (ACE) inhibitors, angiotensin II receptor blockers (ARBs), beta-blockers, diuretics, erythropoietin-stimulating agents (ESA), and calcium-based phosphate binders. The type of vascular access was confirmed using procedure codes from claims data within 360 days prior to or 180 days after hemodialysis initiation. The facility where the patient underwent regular maintenance hemodialysis in the ambulatory care setting, i.e., medical centers, regional hospitals, community hospitals, or clinics and the urbanization levels of the hemodialysis units were recorded.

### Definition of exposure and outcomes

We collected prescription information of calcitriol and alfacalcidol, exclusively oral form, from claim data of ambulatory care and hospital admission. The 360th day after hemodialysis initiation was selected to define patients as vitamin D users or non-users according to whether they were prescribed vitamin D before the landmark time.

The primary outcome of interest included occurrence of AMI, ischemic stroke, amputation, and infection hospitalization, respectively. Because a patient may die before experiencing the first event of interest, this death event is a competing risk and precludes the occurrence of the event of interest [18].

Considering the genomic effects of vitamin D which may not be immediate, patients who died or were admitted due to these events before the landmark date were excluded. This design helps to evaluate the legacy effect of activated vitamin D and avoid potential for immortal time bias [19, 20].

For hospital admission claims, up to 5 discharge diagnoses would be documented to the NHI for reimbursement. The most important diagnoses or billable codes would be listed first. We recorded the first occurrence of hospitalization due to AMI (ICD-9 code: 410), ischemic stroke (ICD-9 codes: 433, 434), amputation (procedure code: 841), and infection (ICD-9 codes: 480–488, 590, 595, 601, 604, 608.83, 680–682, 685, 686, 038, 728.86, 790.7, 040.0, 785.4, 995.91, 995.92, 996, 999.31–999.33) after landmark date, respectively (Table S1).

### Statistical analyses

For baseline characteristics, continuous variables were presented as mean (standard deviation, SD) with categorical variables as number (percentage) (Table 1). Comparisons between vitamin D users and non-users were made by calculating standardized difference (*d*) for which the value of less than 0.10 indicates a negligible difference [21].

**Table 1:** Baseline characteristics of activated vitamin D users versus non-users according to status by landmark time

	**Vitamin D users**	**Non-users**	d*
**N (%)**	8151 (15.5)	44,606 (84.5)	
**Age, year**	58.9 (14.1)	62.5 (13.3)	0.26
**< 53**	2847 (34.9)	10,949 (24.6)	0.25
**≥ 53 and < 64**	2128 (26.1)	11,653 (26.1)	
**≥ 64 and < 73**	1749 (21.5)	11,325 (25.4)	
**≥ 73**	1427 (17.5)	10,679 (23.9)	
**Gender (male)**	3680 (45.2)	22,619 (50.7)	0.11
**Comorbidities**			
**DM**	3327 (40.8)	26,616 (59.7)	0.38
**CHF**	2200 (27.0)	15,195 (34.1)	0.15
**MI**	1932 (23.7)	13,868 (31.1)	0.17
**PVD**	259 (3.2)	1509 (3.4)	0.01
**CVD**	774 (9.5)	7095 (15.9)	0.19
**COPD**	14 (0.2)	128 (0.3)	0.02
**CTD**	176 (2.2)	1021 (2.3)	< 0.01
**PUD**	1344 (16.5)	8023 (18.0)	0.04
**Neoplasia**	10 (0.1)	56 (0.1)	< 0.01
**Chronic liver diseases**	1001 (12.3)	5353 (12.0)	< 0.01
**Vascular access type**			0.15
**AVF**	6372 (78.2)	34,240 (76.7)	
**AVG**	617 (7.6)	4308 (9.7)	
**Permanent catheter**	116 (1.4)	1097 (2.5)	
**Double lumen catheter**	539 (6.6)	3219 (7.2)	
**Unknown**	507 (6.2)	1742 (3.9)	
**Hospital level**			0.42
**Medical centers**	1683 (20.7)	4006 (8.9)	
**Regional hospitals**	2499 (30.7)	10,462 (23.5)	
**Community hospitals**	1267 (15.5)	8777 (19.7)	
**Clinics**	2702 (33.1)	21,361 (47.9)	
**Urbanization level**			0.35
**Urban**	4293 (52.7)	15,976 (35.8)	
**Suburban**	1048 (12.9)	6453 (14.5)	
**Rural**	108 (1.3)	816 (1.8)	
**Unknown**	2702 (33.1)	21,361 (47.9)	
**Medications**			
**Antiplatelets**	3929 (48.2)	24,796 (55.6)	0.15
**Aspirin / Clopidogrel**	2324 (28.5)	15,600 (35.0)	0.14
**Cilostazol**	154 (1.9)	1146 (2.6)	0.05
**Warfarin**	143 (1.8)	988 (2.2)	0.03
**Statins**	1373 (16.8)	9535 (21.4)	0.12
**Insulin**	1615 (19.8)	12,898 (28.9)	0.21
**OAD**	1812 (22.2)	16,003 (35.9)	0.30
**Metformin**	179 (2.2)	1857 (4.2)	0.11
**Sulfonylurea**	917 (11.3)	8041 (18.0)	0.19
**α-glucosidase inhibitors**	148 (1.8)	1478 (3.3)	0.09
**TZD**	81 (1.0)	684 (1.5)	0.05
**DPP-4 inhibitors**	2 (0.02)	5 (0.01)	0.01
**Meglitinides**	485 (6.0)	3938 (8.8)	0.11
**ACEI / ARB**	3972 (48.7)	23,726 (53.2)	0.09
**Beta-blockers**	4173 (51.2)	24,243 (54.4)	0.06
**Diuretics**	5737 (70.4)	34,377 (77.1)	0.15
**ESA**	1887 (23.2)	10,133 (22.7)	0.01
**Ca-based P-binders**	5027 (61.7)	20,321 (45.6)	0.32

Note:

(1) The landmark time is the 360th day of initiation of hemodialysis

(2) Values for categorical variables are given as numbers (percent); for continuous variables, as means (standard deviation)

*Abbreviations*: DM, diabetes mellitus; CHF, congestive heart failure; MI, myocardial infarction; PVD, peripheral vascular disease; CVD, cerebrovascular disease; COPD, chronic obstructive pulmonary disease; CTD, connective tissue disease including rheumatoid arthritis, systemic lupus erythematosus, etc.; PUD, peptic ulcer disease; Chronic liver diseases: chronic viral hepatitis, cirrhosis and its complications; AVF: arteriovenous fistula; AVG: arteriovenous graft

OAD, oral antidiabetic drugs; TZD, thiazolidinediones; DPP-4 inhibitors, dipeptidyl peptidase 4 inhibitors; ACEI / ARB, angiotensin converting enzyme inhibitors/angiotensin II receptor blockers; ESA, erythropoiesis-stimulating agents; Ca-based P-binders, calcium-based phosphate binders

* Standardized difference (*d*): statistically significantly different between two comparison groups if *d* > 0.10

Crude incidence rates for death, and events of interest were calculated by dividing the number of events by the cumulative at-risk time. We performed competing risk analysis primarily using the subdistribution hazard function, also known as Fine-Gray model, to estimate the subdistribution hazard ratios (SHRs) of various events of interest (e.g., AMI) and the associated 95% confidence interval (CI) in association with vitamin D use. Death before the event of interest was considered as a competing event. We then used cause-specific hazard models to confirm the association observed in the primary analysis. The difference between the two competing risk models is that patients who have experienced a competing event are retained in the risk set of subdistribution hazard model, but are excluded in the cause-specific hazard model [22]. Age, sex, vascular access type, comorbidities, baseline medications, the hospital accreditation level and the urbanization level where the hemodialysis units located were included in the models as potential confounders. We repeated the above analyses for AMI, ischemic stroke, amputation, and infection hospitalization as events of interests, respectively. We plotted and compared cumulative incidence curves among vitamin D users and non-users using cumulative incidence competing risk method.

For possible under-estimation of AMI incidence given the inherently elevated cardiac enzymes in ESRD patients and potentially neglected by physicians, we conducted several sensitivity analyses. First, we incorporated patients with acute coronary syndrome or unstable coronary artery diseases (ICD-9 code: 411). Second, we incorporated patients with AMI diagnoses in emergency rooms to avoid missing those who were not admitted eventually. Third, we concerned that the independent censoring assumption of competing risk analysis is violated and thus performed two additional analyses. We first considered that all subjects censored because of “deaths” are assumed to have events of interest, i.e. AMI, instead. Afterward, we randomly selected a subset of 50% of subjects censored to death and then assumed that everyone in the subset has AMI and repeated the analysis.

We also performed prespecified subgroup analyses according to sex, age, the presence or absence of major cardiovascular risk factors, and hospital accreditation and urbanization levels as a surrogate of socioeconomic status. Regarding infection as event of interest, we further included the presence or absence of chronic liver disease and chronic pulmonary disease in the subgroup analyses. Within each level of the stratified variable, the analysis was multivariate adjusted for all potential confounders.

Finally, we added the variable “dosage group” obtained from the trajectory analysis in our previous study into the multivariate competing risk model to evaluate if dose response existed [17].

All statistical analyses were conducted using SAS version 9.4 (SAS Institute Inc., Cary, NC).

## Results

We identified 61,485 uremic patients who had initiated hemodialysis from 2001 to 2009 and had been issued CIC for dialysis dependence. Of these, we excluded those who died (*n* = 5757) or had follow-up of less than 360 days (*n* = 2921). Finally, 52,757 patients were included and were followed for a median of 1019 (interquartile range, 473–1888) days from the landmark date. According to vitamin D prescription or not before the landmark date, there were 8151 vitamin D users and 44,606 non-users. Vitamin D users were younger than non-users and had fewer baseline cardiovascular comorbidities (Table 1).

A total of 2723 patients were hospitalized for AMI, 3460 had ischemic stroke, 2623 experienced an amputation procedure, and 22,082 patients hospitalized for infection after the landmark date, respectively. The numbers of competing events of death differ between individual events of interest, respectively (Table 2).

**Table 2:** Incidence rates of acute myocardial infarction, ischemic stroke, amputation, and hospitalization for infection after landmark date (the 360th day) and competing events of death for vitamin D users versus non-users, respectively

	**Follow-up (person-year)**	**Event of interest (%)**	**Event of interest** **(per 1000 p-y)**	**Death (%)**^**a**^	**Crude death rate** **(per 1000 p-y)**	**Total**
**AMI**						
**Non-user**	137,639.35	2364 (5.4)	17.2	16,312 (37.5)	118.5	43,495
**Vitamin D user**	28,378.86	359 (4.5)	12.6	2340 (29.3)	82.5	8020
**All**	166,018.21	2723 (5.3)	16.4	18,652 (36.2)	112.3	51,515
**Ischemic stroke**						
**Non-user**	135,591.67	3045 (7.5)	22.5	15,936 (39.4)	117.5	40,407
**Vitamin D user**	28,133.93	415 (5.2)	14.8	2312 (28.8)	82.2	8022
**All**	163,725.60	3460 (6.7)	21.1	18,248 (35.5)	111.5	51,429
**Amputation**						
**Non-user**	138,462.73	2370 (5.4)	17.1	16,629 (37.7)	120.1	44,105
**Vitamin D user**	28,735.85	253 (3.1)	8.8	2426 (29.9)	84.4	8109
**All**	167,198.58	2623 (5.0)	15.7	19,055 (36.5)	113.9	52,214
**Infection**						
**Non-user**	97,982.67	19,178 (50.4)	195.7	4513 (11.8)	46.1	38,075
**Vitamin D user**	21,014.29	2904 (41.0)	138.2	759 (10.7)	36.1	7079
**All**	118,996.96	22,082 (48.9)	185.5	5272 (11.7)	44.3	45,154

^a^Note: Death is a competing event that precludes the occurrence of events of interest, including AMI, ischemic stroke, amputation, and infection, respectively

*Abbreviations*: AMI, acute myocardial infarction; p-y, person-year

**Fig. 1:**
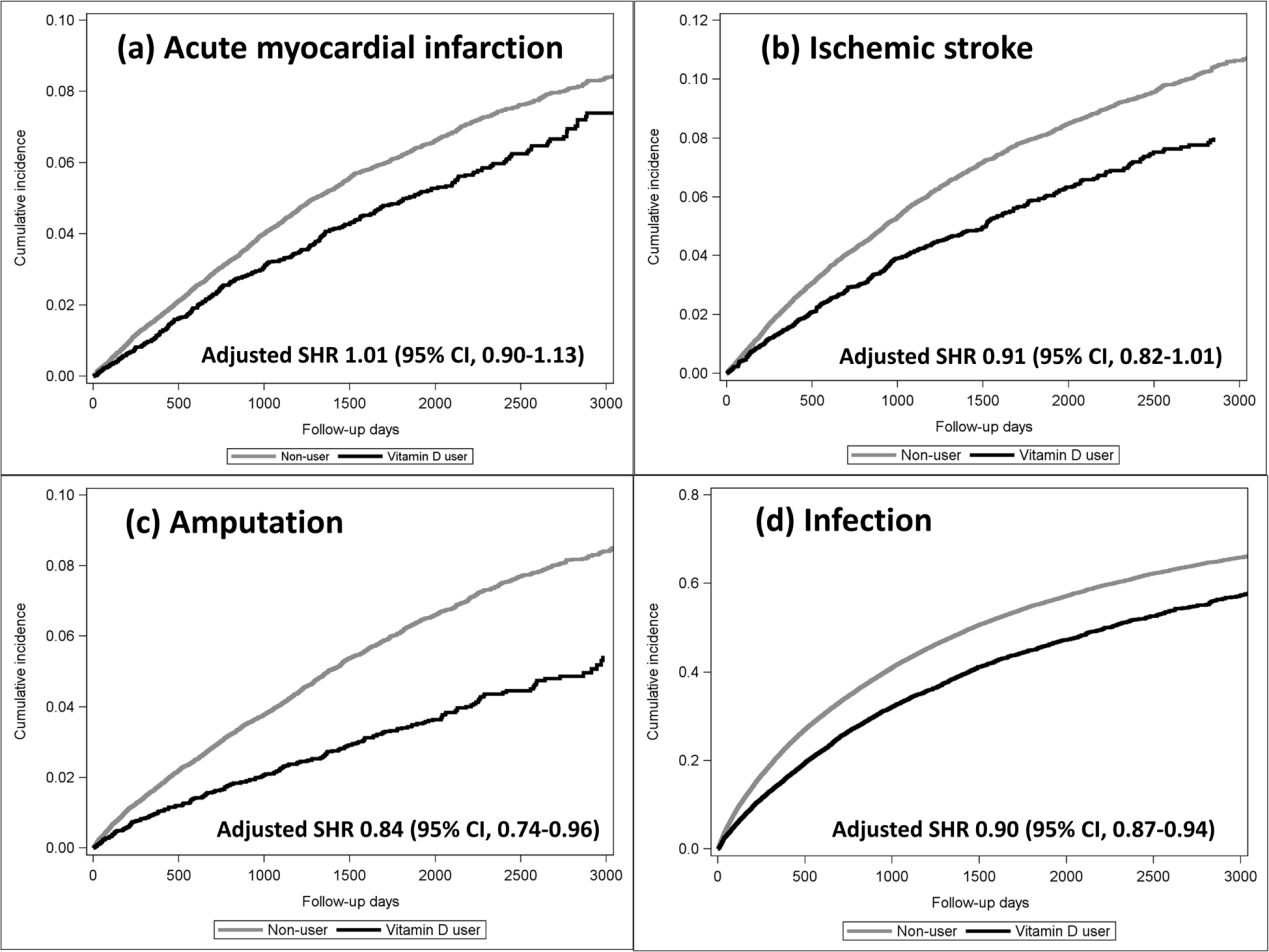
Cumulative incidence curves of acute myocardial infarction (AMI), ischemic stroke, amputation procedure, and hospitalizations for infection for vitamin D users compared with non-users. **a** AMI (left upper panel) **b** Ischemic stroke (right upper panel) **c** amputation (left lower panel) **d** infection (right lower panel). Taking death as competing events into consideration, vitamin D users have lower cumulative incidences of AMI, ischemic stroke, amputation, and infection, compared with non-users

Vitamin D users had a lower incidence rate of AMI than non-users (12.6 versus 17.2 per 1000 person-years) and fewer competing events of death (112.3 versus 118.5 per 1000 person-years). Similarly, the incidence rates of ischemic stroke, amputation, hospitalization for infection, and competing events of death were all lower for vitamin D users than that in non-users (Table 2). Cumulative incidence curves for each event of interest are shown (Fig. 1).

Taking into consideration competing event of death, the estimated SHR for AMI was 0.82 (95% CI, 0.73–0.91) in unadjusted and 1.01 (95% CI, 0.90–1.13) in fully-adjusted model for vitamin D users compared to non-users, respectively (Table 3). The unadjusted and multivariable adjusted SHR of ischemic stroke were 0.73 (95% CI, 0.66–0.81) and 0.90 (95% CI, 0.81–1.01) for vitamin D users compared with non-users, respectively. With death as a competing event, the multivariable adjusted SHR of amputation and infection hospitalization were 0.84 (95% CI, 0.74–0.96) and 0.91 (95% CI, 0.88–0.95) for vitamin D users compared to non-users.

**Table 3:** The hazard of acute myocardial infarction (AMI), ischemic stroke, amputation, and hospitalization for infection for vitamin D users compared with non-users in incident hemodialysis patients using competing risk analysis with subdistribution hazard model and cause-specific hazard regression models

	**AMI**	**Ischemic stroke**	**Amputation**	**Infection**
	**SHR (95% CI)**	**SHR (95% CI)**	**SHR (95% CI)**	**SHR (95% CI)**
**Model 1**	0.82 (0.73–0.91)	0.73 (0.66–0.81)	0.57 (0.50–0.65)	0.75 (0.72–0.78)
**Model 2**	0.89 (0.79–0.99)	0.78 (0.70–0.87)	0.58 (0.51–0.66)	0.82 (0.78–0.85)
**Model 3**	1.03 (0.92–1.15)	0.91 (0.82–1.01)	0.82 (0.72–0.95)	0.90 (0.87–0.94)
**Model 4**	1.04 (0.93–1.16)	0.92 (0.83–1.02)	0.84 (0.74–0.96)	0.91 (0.87–0.94)
**Model 5**	1.04 (0.93–1.16)	0.91 (0.82–1.01)	0.84 (0.74–0.96)	0.91 (0.88–0.95)
**Model 6**	1.01 (0.90–1.13)	0.91 (0.82–1.01)	0.84 (0.74–0.96)	0.90 (0.87–0.94)
**Model 7**	1.01 (0.90–1.13)	0.90 (0.81–1.01)	0.84 (0.74–0.96)	0.91 (0.88–0.95)
	**CSHR (95% CI)**	**CSHR (95% CI)**	**CSHR (95% CI)**	**CSHR (95% CI)**
**Model 1**	0.74 (0.66–0.83)	0.66 (0.60–0.73)	0.52 (0.45–0.59)	0.72 (0.69–0.75)
**Model 2**	0.83 (0.74–0.92)	0.73 (0.66–0.81)	0.55 (0.48–0.62)	0.80 (0.77–0.83)
**Model 3**	0.99 (0.89–1.12)	0.89 (0.80–0.98)	0.79 (0.70–0.90)	0.90 (0.87–0.94)
**Model 4**	1.01 (0.90–1.13)	0.90 (0.81–0.99)	0.81 (0.71–0.93)	0.91 (0.88–0.95)
**Model 5**	1.01 (0.90–1.13)	0.89 (0.80–0.99)	0.82 (0.72–0.93)	0.92 (0.88–0.95)
**Model 6**	0.99 (0.88–1.11)	0.90 (0.81–0.99)	0.82 (0.72–0.94)	0.91 (0.87–0.95)
**Model 7**	0.99 (0.89–1.12)	0.90 (0.81–0.99)	0.83 (0.73–0.94)	0.92 (0.88–0.96)

Model 1: unadjusted crude SHR. Model 2: adjusted for age and sex. Model 3: model 2 plus comorbidities

Model 4: model 3 plus medication use. Model 5: model 4 plus vascular access type. Model 6: model 5 plus hospital and urbanization levels

Model 7: model 6 plus use of calcium-based phosphate binders

SHR: subdistribution hazard ratio. CI: confidence interval. CSHR: cause-specific hazard ratio

For possible underestimation of AMI incidence, we extended the definition of AMI and repeated the analysis. There were 4661 patients admitted for AMI or unstable angina while 17,446 had competing events of death. Vitamin D users did not have a lower risk of AMI (SHR 1.04 [95% CI, 0.96–1.14]), compared with non-users. We further incorporated patients diagnosed AMI in the emergency room into the analysis. The results were essentially similar.

Considering that the assumption that censoring is unrelated to the outcome events may have been violated, we assumed that all subjects censored because of deaths (*n* = 18,048) have events of interest of AMI instead. In this extreme scenario, vitamin D users were found to have a lower risk of AMI (SHR 0.92 [95% CI, 0.89–0.96]). If half of the patients censored because of death were attributed to AMI, vitamin D users were also associated with a lower risk of AMI, compared with non-users (SHR 0.94 [95% CI, 0.89–0.99]).

We performed stratified analyses to evaluate the existence of interaction between baseline covariates and activated vitamin D use on the outcome of amputation and infection, respectively. The choice of baseline covariates in the interaction terms included age, sex, comorbidities, hospital accreditation level and urbanization which could be potentially associated with amputation or infection occurrence, according to clinical experience and evidences from the literature review [23–25].

**Fig. 2:**
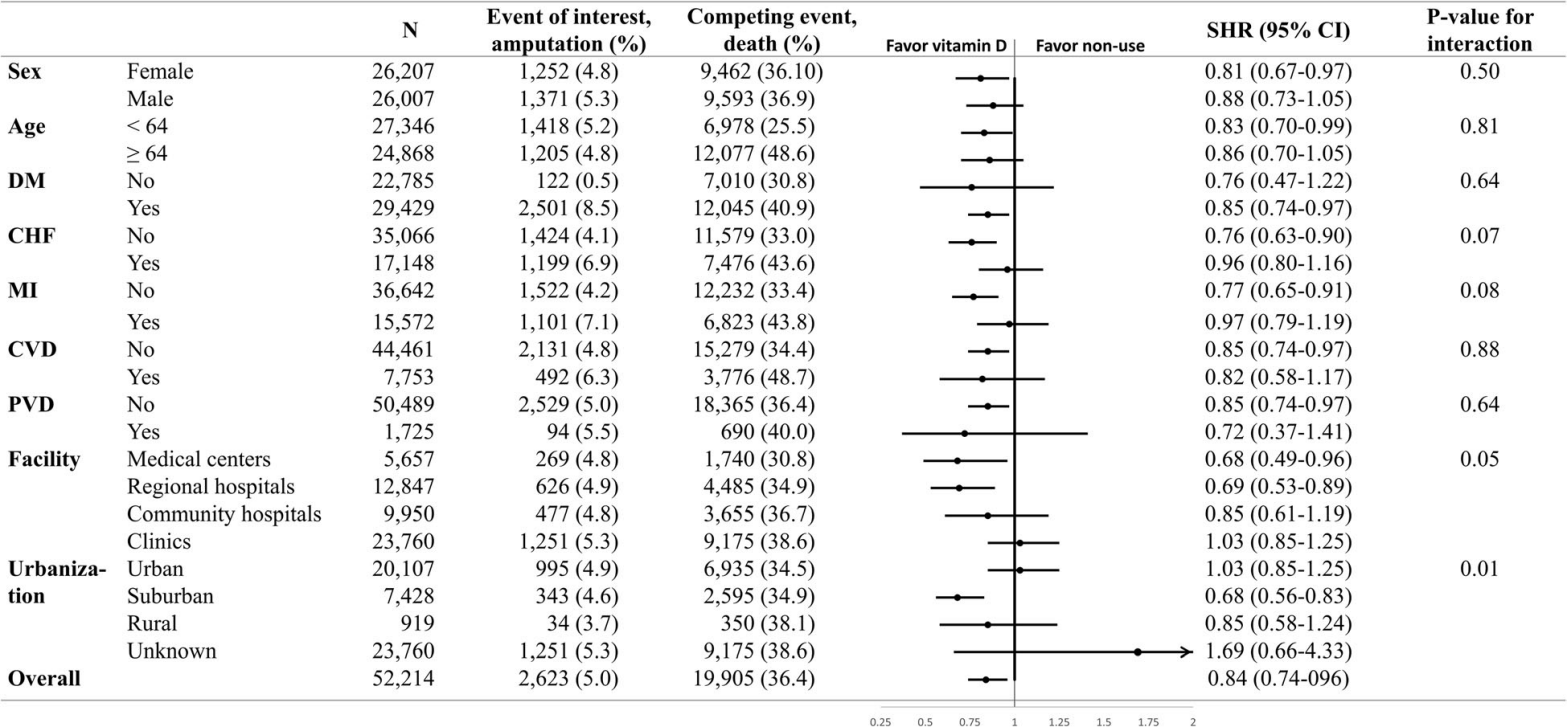
Forest plot shows the associated subdistribution hazard ratios of activated vitamin D users compared with non-users on the outcomes of interest (amputation), according to baseline characteristics. *Abbreviations*: DM, diabetes mellitus; CHF, congestive heart failure; MI, myocardial infarction; CVD, cerebrovascular disease; PVD, peripheral vascular disease. SHR, subdistribution hazard ratio; CI, confidence interval

We found that the associated hazards of amputation were reduced substantially in subgroups of females, patients aged less than 64 years, those with comorbidities of diabetes, and those without history of congestive heart failure, myocardial infarction, cerebrovascular disease, or peripheral vascular disease but not significantly in their counterparts (Fig. 2). In addition, the hazard of amputation associated with activated vitamin D use were also lower in those who had maintenance hemodialysis treatment in the medical centers or regional hospitals. Nevertheless, the subdistribution hazard ratios across these subgroups were nearly all in the same direction, suggesting that the potential impact of activated vitamin D use on the incidence of amputation was not significantly modified by age, sex, diabetes or baseline cardiovascular comorbidities.

**Fig. 3:**
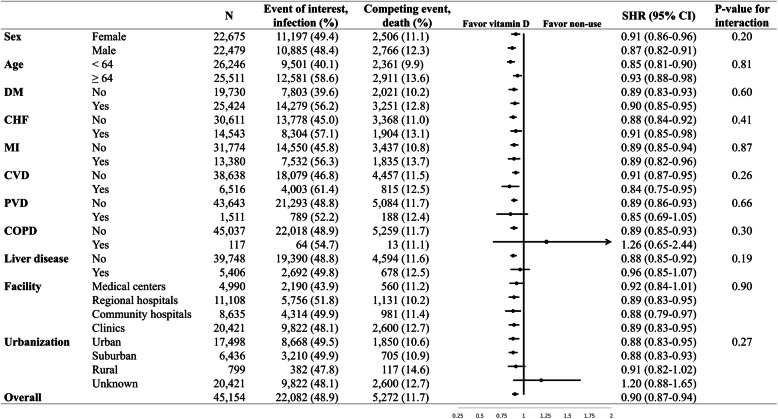
Forest plot shows the associated subdistribution hazard ratios of activated vitamin D users compared with non-users on the outcomes of interest (infection), according to baseline characteristics. *Abbreviations*: DM, diabetes mellitus; CHF, congestive heart failure; MI, myocardial infarction; CVD, cerebrovascular disease; PVD, peripheral vascular disease. SHR, subdistribution hazard ratio; CI, confidence interval

For infection as the event of interest, there was no evidence of heterogeneity in the result for any subgroup evaluated (Fig. 3). The reduced subdistribution hazards of infection favoring activated vitamin D treatment were observed in nearly every subgroup, including subgroups according to age, gender, and status of chronic comorbidities such as diabetes, cerebrovascular disease, and heart disease.

We further performed stratified analyses for chronic obstructive pulmonary disease (COPD) and cirrhosis for these may contribute to increased risk of infection. The association of activated vitamin D and the incidence of infection was still not modified by these two comorbidities. Though the estimated risk of infection was not decreased in vitamin D users compared to non-users among those with COPD, the wide confidence interval may imply the imprecise estimate from the scarce patient number identified in the claim database during the baseline period.

We had utilized trajectory analysis by evaluating the cumulative dosage of vitamin D in three 120-day intervals in the first 360 days of hemodialysis initiation. Vitamin D users were thus classified into two groups. Using 0.25 microgram as one dosage unit, the majority of vitamin D users (*n* = 6849) received an average of 67.5, 45.2, and 43.1 dosage units, while high dose vitamin D users (*n* = 326) received 750.4, 68.9, and 53.1 dosage units in the three 120-day interval, respectively. With non-users as the reference group, we analyzed if a dose response existed in the outcome of amputation and infection, respectively. The estimated risk of amputation was SHR 0.83 (95% CI, 0.72–0.96) for conventional dose vitamin D users and SHR 0.52 (95% CI, 0.17–1.63) for high dose users. By contrast, the estimated hazard of infection was SHR 0.90 (95% CI, 0.86–0.94) for conventional dose users and SHR 0.69 (95% CI, 0.57–0.85) for high dose users compared with non-users (Table 4).

**Table 4:** Multivariate adjusted competing risk model for events of amputation and infection according to dosage of activated vitamin D based on trajectory analysis

	**N (%)**	**Events of interest (%)**	**Death (%)**	**SHR (95% CI)**
**Amputation**				
**Non-user**	45,069 (86.3)	2410 (5.4)	17,019 (37.7)	Reference
**Conventional dose user**	6820 (13.1)	210 (3.1)	1963 (28.8)	0.83 (0.72–0.96)
**High dose user**	325 (0.6)	3 (0.9)	73 (22.5)	0.52 (0.17–1.63)
**Overall**	52,214 (100)	2623 (5.0)	19,055 (36.5)	0.84 (0.74–0.96)
**Infection**				
**Non-user**	38,860 (86.1)	19,586 (50.4)	4614 (11.9)	Reference
**Conventional dose user**	5997 (13.3)	2406 (40.1)	628 (10.5)	0.90 (0.86–0.94)
**High dose user**	297 (0.7)	90 (30.3)	30 (10.1)	0.69 (0.57–0.85)
**Overall**	45,154 (100)	22,082 (48.9)	5272 (11.7)	0.91 (0.88–0.95)

The competing risk model (Fine-Gray) was adjusted by covariates including age, sex, vascular access type, baseline comorbidities, and medications

*Abbreviations*: *SHR* subdistribution hazard ratio, *CI* confidence intervals

## Discussion

Taking competing events of death into consideration, we found that vitamin D users were significantly associated with a lower risk of amputation or hospitalization due to infection, but not AMI or ischemic stroke than those not treated within 360 days of hemodialysis initiation. Though cardiovascular disease is one of the leading causes of death in ESRD patients, prescription of activated vitamin D does not seem to be directly associated with a reduced risk of AMI or ischemic stroke in these patients.

Both atherosclerosis and arteriosclerosis contributes to cardiovascular morbidity or mortality in ESRD patients. The former comes from lipid and macrophage infiltration, formation of atheroma in the arterial intima causing vascular narrowing and/or plaque rupture. The later comes from vitamin D deficiency, dysregulated mineral metabolism, and vascular medial calcification [26]. Long-term complications of vascular calcification include impaired myocardial relaxation, heart failure, and peripheral vascular disease [6]. Evidences had suggested that old age, diabetes and cardiovascular disease were directly associated and 25-hydroxyvitamin D levels were inversely associated with vascular calcifications [27]. In patients with symptomatic PAD, those with higher calcification scores in the lower extremity arteries predicted worse outcomes, including amputation and mortality [28]. Prescription of activated vitamin D may bring about the potential benefit of vitamin D replacement and to reverse or retard the vascular calcification process [29]. This may translate into a reduced risk of lower limb amputation.

It is possible that the true incidence of AMI is higher than estimated (5.28%) in our cohort. However, a similar percentage (5.44%) of AMI was found in the historical cohort of 627,983 patients in the United States Renal Data System (USRDS) from 1977 to 1995 [30]. Assuming that half of the competing events of death may be related to AMI or its complications such as heart failure or fatal arrhythmia though overlooked, activated vitamin D use may be beneficial for cardiovascular outcomes.

Infection or septicemia are second to cardiovascular disease as the leading cause of death in ESRD patients [31]. Of them, infections associated with vascular access, soft tissue infection, and pneumonia are the most common sources [32]. In our cohort, the most common kind of infection is pneumonia (52.8%), followed by infection related to implant, catheter, or prosthesis (22.4%), and soft tissue or subcutaneous infection (19.3%). More than half (56.9%) would be designated as sepsis. Costs associated with infection events pose a significant economic burden to the health care system.

Findings from previous studies have suggested a reduced risk of respiratory infection or infection-related death in ESRD patients receiving activated vitamin D [16, 33]. There were conflicting evidences that vitamin D was not effective in prevention of hospitalization for infection [34]. The different conclusions from these studies may stem from the study design and the age of the cohort participants. There seems no benefit of activate vitamin D observed in the reduction of infection-related hospitalization in the Canadian cohort with older age (mean 68 years) [34]. In contrast, among the cohort recruited from a single center in Japan (mean age 59.6 years), a lower risk of respiratory tract infection was observed among vitamin D users compared to non-users [16]. Conclusions drawn from our study incorporating extensive kinds of infection not confined to respiratory or urinary tract infection in the real world setting support the benefit of reduction of infection hospitalization from vitamin D treatment.

The interpretation of the two commonly used methods for competing risk analyses differs. Cause-specific hazard regression is more suitable for etiology inference since it focuses on the event of interest and treats competing events as censored. In contrast, the subdistribution hazard model is more fitted for prognostic prediction because it takes into account the competing events in the risk set for which the censoring may not be independent [35, 36]. Under the circumstances that the independent censoring assumption is violated, the effect estimate obtained from subdistribution hazard regression may be more reliable. In our study, the associated effect estimates of vitamin D use on the hazards of AMI, ischemic stroke, amputation, and infection were mostly in the same direction in the two competing risk models.

The result of the stratified analyses showed that female gender, younger, and diabetic patients were associated with significant benefits of vitamin D for a reduced hazard of amputation. By contrast, the potential associated impact of activated vitamin D on the incidence of infection was homogenous across these subgroups. Overall, the association between of activated vitamin D use and the reduced incidence of amputation and infection were essentially not modified by sex or baseline comorbidities.

For vitamin D users, those receiving higher dose were associated with a significantly lower subdistribution hazard of infection (*p*-value for trend < 0.0001). By contrast, despite that only 3 out of the 325 patients in the high dose group experienced amputation made the effect estimate statistically insignificant, there was still a positive dose gradient for the reduced subdistribution hazard of amputation (*p*-value for trend 0.02).

The limitations of our study include that we have no data about calcium, phosphorus, and parathyroid hormone levels which may influence the overall outcomes. We had no information about the use of calcimimetics or paricalcitol, though they were not frequently prescribed due to the expensive price. In addition, we used vitamin D prescription to define vitamin D use, which might be subject to information error. Besides, diagnostic codes for heart failure are not validated and cannot be adopted as event of interest. The fact that activated vitamin D use is associated with a significantly lower risk of death may have come from the benefit from decreasing the risk of heart failure or its complications which were not readily identified in the claim database.

Finally, because we included multiple endpoints in the analysis, which is subjected to a concern about the problem of multiple testing, which in turn may produce inflation of type 1 error [37]. Therefore, interpretation of the findings from our study should proceed with caution.

## Conclusion

Use of activated vitamin D is associated with lower risks of amputation and infection hospitalization. The associated effect of activated vitamin D with a reduced risk of infection is consistent across all subgroups while the advantage to lower risk of amputation seems strengthened in female, younger, diabetic, and otherwise healthy patients.

Lower limb amputation, which implies peripheral vascular disease, coincides with and is representative of heart failure and cardiovascular morbidities, contribute to high mortality in hemodialysis patients. Activated vitamin D use in these patients may bring about the benefits of decreasing infection events or amputation, which is a complication of peripheral vascular disease, rather than reducing major atherosclerotic events such as AMI or ischemic stroke. Future studies evaluating vitamin D use and surrogate markers or outcomes for heart failure or immune function may be anticipated.

## Supplementary information

**Additional file 1: Table S1.** ICD-9 codes for AMI, ischemic stroke, and various kinds of infection.

## Data Availability

The claims databases of health insurance can be accessed with the permission of the Ministry of Health and Welfare, Taiwan. The analytical data derived from the databases relevant to the study are available from the corresponding author on reasonable request.

## Abbreviations

NHIRD: National Health Insurance Research Database
AMI: Acute myocardial infarction
SHR: Subdistribution hazard ratio
CKD: Chronic kidney disease
ESRD: End-stage renal disease
PAD: Peripheral artery disease
CIC: Catastrophic illness certificate
ICD-9-CM: International Classification of Disease, 9th revision, Clinical Modification
CSHR: Cause-specific hazard ratio
