# Supplementary material for: The use of activated vitamin D and risks of hospitalization for infection and amputation in incident hemodialysis patients in Taiwan: a nationwide population-based cohort study
Source: BMC Nephrol. 2020 Aug 6;21:331. doi: 10.1186/s12882-020-01988-2 (PMC7409709; doi:10.1186/s12882-020-01988-2)
Supplement: Supplementary file 1 — Additional file 1: Table S1. ICD-9 codes for AMI, ischemic stroke, and various kinds of infection. [file 12882_2020_1988_MOESM1_ESM.docx]

**The use of activated vitamin D and risks of hospitalization for infection and amputation in incident hemodialysis patients in Taiwan: A nationwide population-based cohort study**

**Table S1. ICD-9 codes for AMI, ischemic stroke, and various kinds of infection**

| **ICD-9 code** | |
| --- | --- |
| **Acute myocardial infarction** | |
| 410 | Acute myocardial infarction |
| 411 | Other acute and subacute forms of ischemic heart disease |
| **Ischemic stroke** | |
| 433 | Occlusion and stenosis of precerebral arteries |
| 434 | Occlusion of cerebral arteries |
| **Infection** | |
| 480-488 | Pneumonia |
| 590 | Infections of kidney |
| 595 | Cystitis |
| 601 | Prostatitis |
| 604 | Orchitis and epididymitis |
| 608.83 | Fournier gangrene |
| 680 | Carbuncle and furuncle |
| 681 | Cellulitis and abscess of finger and toe |
| 682 | Other cellulitis and abscess |
| 685 | Pilonidal cyst |
| 686 | Other local infections of skin and subcutaneous tissue |
| 038 | Septicemia |
| 790.7 | Bacteremia |
| 728.86 | Necrotizing fasciitis |
| 040.0 | Gas gangrene |
| 785.4 | Gangrene |
| 995.91, 995.92 | Sepsis |
| 996.6 | Infection and inflammatory reaction due to internal prosthetic device implant and graft |
| 996.60 | Infection and inflammatory reaction due to unspecified device implant and graft |
| 996.61 | Infection and inflammatory reaction due to cardiac device implant and graft |
| 996.62 | Infection and inflammatory reaction due to vascular device implant and graft |
| 996.64 | Infection and inflammatory reaction due to indwelling urinary catheter |
| 996.65 | Infection and inflammatory reaction due to other genitourinary device implant and graft |
| 996.66 | Infection and inflammatory reaction due to internal joint prosthesis |
| 996.67 | Infection and inflammatory reaction due to other internal orthopedic device implant and graft |
| 996.69 | Infection and inflammatory reaction due to other internal prosthetic device implant and graft |
| 999.31 | Other and unspecified infection due to central venous catheter |
| 999.32 | Bloodstream infection due to central venous catheter |
| 999.33 | Local infection due to central venous catheter |
